# Collecting Long-Term Wearable Data in Older Adults: Atherosclerosis Risk in Communities Neurocognitive Study

**DOI:** 10.1093/geroni/igaf122.3437

**Published:** 2025-12-31

**Authors:** Erin Spaulding, Jie Ding, Henry Zhao, Pamela Lutsey, B Gwen Windham, Katherine Ornstein, Josef Coresh, Seth Martin

**Affiliations:** Johns Hopkins University, Baltimore, Maryland, United States; Johns Hopkins University School of Medicine, Baltimore, Maryland, United States; Johns Hopkins University School of Medicine, Baltimore, Maryland, United States; University of Minnesota, Minneapolis, Minnesota, United States; University of Mississippi Medical Center - The MIND Center, Jackson, Mississippi, United States; Johns Hopkins University, Baltimore, Maryland, United States; New York University, New York, New York, United States; Johns Hopkins University School of Medicine, Baltimore, Maryland, United States

## Abstract

Traditional cohort studies, relying on periodic visits, may introduce bias by capturing data largely when participants feel well. Wearables enable continuous data collection, potentially mitigating this bias, but low adoption among older adults could result in data gaps. Fitbit Charge 6 devices were used to collect long-term health data among Atherosclerosis Risk in Communities Neurocognitive Study (ARIC NCS) participants with a smartphone in 2024-2025. Staff provided Fitbit setup and training, encouraging daily wear and regular syncing, with ongoing technology support. Fitbit wear time, measured by heart rate data, was calculated as a percentage. Mann-Whitney U tests assessed differences in wear time by age, sex, and race. Approximately one-third of eligible participants offered a Fitbit chose to enroll. A total of 309 ARIC NCS participants (84.0±3.2 years [36% ≥85 years], 60% women, 82% White, 45% ≤high school/vocational school) contributed a median of 125 (IQR: 148) days of Fitbit data (∼4.2 months). In this sample of older adults aged 79-95 years, the median wear time was 99% (IQR: 3%). A Mann-Whitney U test revealed a significant difference (z=-4.08, p < 0.001) in median wear time between Black (98%, IQR: 10%) and White (99%, IQR 3%) older adults, indicating greater variability in wear time among Black adults. These findings suggest that with the proper onboarding and ongoing technology support, wearable devices can be used to collect long-term data among adults 80 years and older. Future research could examine potential reasons for greater wear time variability among Black adults, including digital literacy and sensor accuracy.

